# Time-Effect Comparative Evaluation of Three Remineralizing Agents on Artificial Enamel Lesions: A SEM-EDX In Vitro Study

**DOI:** 10.3390/jcm14207389

**Published:** 2025-10-19

**Authors:** Giulia Orilisi, Riccardo Monterubbianesi, Flavia Vitiello, Vincenzo Tosco, Maria Laura Gatto, Paolo Mengucci, Giovanna Orsini

**Affiliations:** 1 Department of Clinical Sciences and Stomatology (DISCO), Università Politecnica delle Marche, 60126 Ancona, Italy; g.orilisi@staff.univpm.it (G.O.); r.monterubbianesi@staff.univpm.it (R.M.); v.tosco@staff.univpm.it (V.T.); g.orsini@staff.univpm.it (G.O.); 2 Department of Industrial Engineering and Mathematical Sciences, Università Politecnica delle Marche, 60131 Ancona, Italy; m.l.gatto@staff.univpm.it; 3 Department of Materials, Environmental Sciences and Urban Planning (SIMAU), Università Politecnica delle Marche, 60131 Ancona, Italy; p.mengucci@staff.univpm.it

**Keywords:** remineralizing agents, dental materials, enamel mineralization, CPP-ACP, F-ACP

## Abstract

**Objective:** This in vitro study quantitatively compared the time-dependent remineralization potential of three professional agents on artificially induced enamel lesions using Scanning Electron Microscopy (SEM) and energy-dispersive X-ray analysis (EDX). **Methods:** Sixty extracted sound molars were randomly assigned to three groups (number = 20): G_CPP-ACP, treated with casein phosphopeptide–amorphous calcium phosphate; G_Zn-HA, treated with zinc-hydroxyapatite; and G_F-ACP, treated with fluoridated amorphous calcium phosphate. The crown of each tooth was divided into three areas: one represented the control (CTRL, sound enamel), one underwent demineralization (DEMIN, demineralized enamel), and the third one was at first demineralized and then treated with a remineralizing agent, allowing intra-sample comparison. Artificial lesions were produced by immersion in 0.1 M lactic acid (72 h). Groups were subdivided according to remineralization time (7, 14, 21, and 28 days). Samples underwent daily treatment under a pH-cycling regimen. Surface morphology and Ca/P ratios were evaluated by SEM-EDX, and data were statistically analyzed (*p* < 0.05). **Results:** All agents promoted a progressive increase in Ca/P ratio over time, confirming a time-dependent remineralization effect. At day 7, G_Zn-HA showed higher Ca/P values, but from day 14 onward, G_F-ACP produced significantly greater mineral gain than the other groups (*p* < 0.05). By day 21, G_F-ACP reached Ca/P values approaching CTRL, while G_CPP-ACP and G_Zn-HA remained at lower levels, reaching a plateau respectively at 21 and 14 days. SEM observations supported these findings: G_CPP-ACP and G_Zn-HA showed partial surface recovery, whereas G_F-ACP exhibited a compact, homogeneous enamel-like structure at 28 days. **Conclusions:** All tested agents demonstrated time-dependent remineralization, enhanced with prolonged exposure, suggesting that the time of application represents a key factor for clinical success.

## 1. Introduction

Dental enamel lesions represent the most prevalent chronic diseases worldwide, and a burden to health-care services [1]. Dental hard tissues are constantly undergoing cycles of demineralization and remineralization [2]. The progression or reversal of such lesions depends on the dynamic equilibrium between demineralization and remineralization occurring in the oral environment, influenced by dietary habits, salivary flow, and preventive interventions [3]. Indeed, a drop in the pH in the oral cavity causes demineralization, which, if it persists for a long period of time, leads to excessive loss of minerals from the tooth structure, resulting in dental enamel lesion. On the contrary, when the pH rises, the reverse takes place, resulting in deposition of mineral, such as calcium, phosphate and fluoride ions, back to the tooth structure, promoting the growth and the formation of new crystals [3].

Nowadays, the best strategy for caries management is to intercept the lesion in its initial state, using methods that improve the remineralizing process with the aid of remineralization products.

The process of tooth mineralization has been extensively studied over many decades, leading to the development of technologies that can either promote enamel remineralization or reduce enamel demineralization, thereby providing potential oral health benefits [4]. Conventional remineralizing strategies have long relied on fluoride-based products, which enhance the precipitation of fluorapatite and improve enamel resistance to acid attack [5]. However, in recent decades, biomimetic remineralizing agents, also associated with fluoride, have emerged as adjunctive or alternative options, aiming to restore enamel structure by mimicking natural mineralization pathways [6,7,8].

Hydroxyapatite (HA), the primary mineral component of dental enamel, has attracted considerable attention as a biomimetic remineralizing agent. Synthetic HA and its modified forms, such as zinc substituted hydroxyapatite (Zn-HA) and nano hydroxyapatite (n-HA), closely mimic the natural crystalline structure of enamel and facilitate mineral deposition onto demineralized surfaces, enhancing remineralization and exerting antibacterial effects due to zinc incorporation [9,10,11].

Furthermore, casein phosphopeptide–amorphous calcium phosphate (CPP-ACP) stabilizes calcium and phosphate ions in a metastable state, promoting their diffusion into enamel subsurface lesions [12,13]. In an acidic environment, ACP separates from CPP, thereby increasing salivary calcium and phosphate levels, and CPP stabilizes the level of ACP in the saliva by preventing precipitation of calcium and phosphate [14]. Recently, fluoridated amorphous calcium phosphate (F-ACP) formulations have been developed, combining the benefits of ACP with fluoride to accelerate the formation of fluorapatite crystals and improve acid resistance of enamel surface [15,16].

Although several studies and systematic reviews have examined these agents individually [17,18,19], comparative data on their time-dependent remineralization potential, particularly integrating morphological and compositional analyses, remain limited.

Scanning electron microscopy (SEM) combined with energy-dispersive X-ray spectroscopy (EDX) offers a robust approach to evaluate surface morphology and quantify mineral content (Ca/P ratio), providing insights into the remineralization process at different stages [20,21].

Despite the growing number of research investigating individual remineralizing agents, there is still a paucity of comparative evidence evaluating their time-dependent performance under standardized experimental conditions. The majority of extant studies have focused on single time points or limited observation periods, without exploring the progressive nature of enamel recovery [4,17]. The present study addresses this gap by performing a longitudinal assessment at four different time intervals (7, 14, 21, and 28 days), thereby providing a dynamic profile of remineralization over time. Furthermore, by integrating SEM with EDX, this investigation combines both morphological and compositional data, thereby facilitating a comprehensive evaluation of enamel surface recovery and mineral gain [22]. The novelty of the present study lies in its multi-time-point SEM-EDX design, which enables the assessment of the progression and trend of the remineralization process over time, providing a dynamic and quantitative understanding of enamel recovery.

At this purpose, the aim of this in vitro study was to quantitatively compare the remineralization potential of agents containing CPP-ACP, Zn-HA, and F-ACP on artificially demineralized enamel lesions over multiple time intervals (7, 14, 21, and 28 days) using SEM-EDX analysis, with sound and demineralized enamel serving as reference controls.

## 2. Materials and Methods

### 2.1. Sample Collection

The study was conducted at the Department of Clinical Sciences and Stomatology of Università Politecnica delle Marche (Ancona, Italy). Teeth were surgically extracted for therapeutic purposes. In accordance with the guidelines of Local Ethical Committee and the Declaration of Helsinki (2018) [23], informed consent was obtained from all patients, ensuring they were fully aware that their hard dental tissues would be used for research purposes. Samples were anonymous at the point of collection and completely unidentifiable in the laboratory.

Following the surgical extraction, the samples underwent a 2 min cleaning in an ultrasonic bath with distilled water, in order to remove blood and biological remains. Teeth were stored in 0.5% *w*/*w* chloramine solution (NH_2_Cl) at room temperature. One single operator performed all procedures to avoid operator bias.

N. 60 extracted sound third molars were selected, meeting the inclusion criteria to minimize sample heterogeneity, as follows: (i) integrity of the buccal and lingual surfaces; (ii) absence of enamel wear, traumatic lesions, restorations, and absence of volume, shape, and structural anomalies; (iii) age of the patients between 20 and 40 years; (iv) no subjected to fluoride treatments.

### 2.2. Samples Preparation and Test Groups

**Figure 1:**
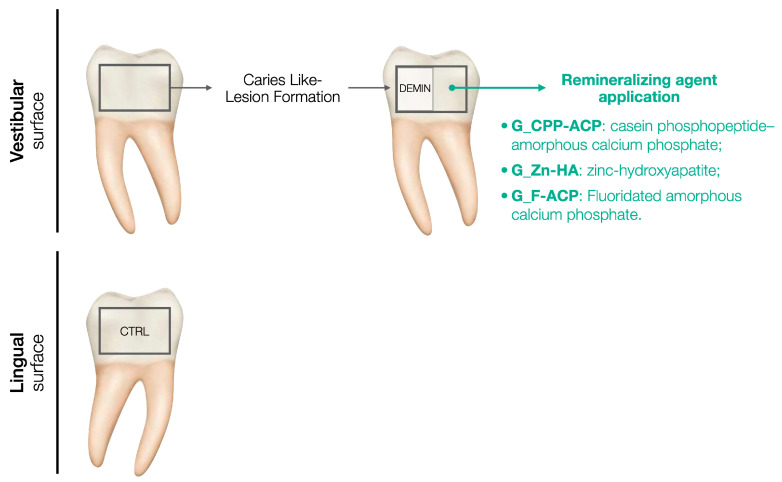
Schematic representation of the study design.

For each sample, the lingual surface was coated with acid-resistant nail varnish, representing the non-demineralized and non-treated enamel (control, CTRL). The buccal surface was demineralized following the caries-like lesion formation protocol and then a half of the surface was coated with acid-resistant nail varnish, indicating the demineralized and not treated enamel (DEMIN). The remaining half buccal surface was subjected to remineralizing agent application according to the group assigned. A schematic representation of the study design is presented in Figure 1.

Teeth were then randomly divided into three groups as follows (n = 20), according to the remineralizing agent used:

- G_CPP-ACP: Treated with a mousse containing CPP-ACP (GC Tooth Mousse, Recaldent GC, Milano, Italy);
- G_Zn-HA: Treated with a gel containing Zn-HA (Biorepair Desensitizing Enamel-Repair Shock Treatment, Coswell oral care professional Spa, Bologna, Italy);
- G_F-ACP: Treated with a mousse containing F-ACP (Biosmalto caries, abrasion and erosion-impact action mousse professional, Curasept Spa, Varese, Italy).

The composition of each agent used in this study is reported in Table 1.

Subsequently, the experimental groups were subdivided into four subgroups, with a total of 5 samples in each subgroup, in accordance with the application time of the remineralization agent:

- Subgroup I—7 days;
- Subgroup II—14 days;
- Subgroup III—21 days;
- Subgroup IV—28 days.

Randomization was performed using a computer-generated randomization list (RAND function, Microsoft Excel).

All the remineralizing agents were applied once a day on the enamel surface for 120 s in a thin layer using a micro brush according to the manufacturer’s instructions.

### 2.3. Caries Like-Lesion Formation

The artificial carious enamel lesion was created on all the samples by immersing the buccal surfaces in a demineralized solution composed of 0.1 M lactic acid adjusted to pH 4.4 using 1 M NaOH for 72 h [20]. To verify lesion formation, each enamel buccal surface was half-covered with a thin layer of nail varnish after the caries like-lesion formation, leaving the other half exposed to the treatment. This procedure allowed direct intra-sample comparison between demineralized and treated region using SEM observation after each time point. Following demineralization, specimens did not undergo passive storage but were immediately treated according to the group assigned and included in a continuous 24 h pH-cycling protocol throughout the experimental period (7, 14, 21, and 28 days).

### 2.4. pH-Cycling Protocol

Samples were subjected to a pH-cycling protocol for the duration of the experiment, in order to simulate pH variations that occur in the oral microenvironment [24,25]. In a 24 h cycle, samples were immersed individually in remineralization solution (1.5 mML-1 calcium, 0.9 mML-1 phosphate, 150 mML-1 potassium chloride in 0.02 mML-1 cacodylic buffer, 0.02 μgF/mL and 1 mL/mm^2^) (pH = 7) for 21 h and immersed in demineralization solution (2.0 mML-1 calcium and phosphate in 75 mML-1 acetate buffer, 0.03 μgF/mL and 3 mL/mm^2^) (pH = 4.3) for 3 h. Samples received the treatment once a day, according to the assigned group. After each treatment, samples were rinsed with deionized water for 5 s. The solutions were replenished every 24 h.

### 2.5. SEM-EDX Analysis

Each subgroup was analyzed according to the time assigned (7, 14, 21 and 28 days). The acid-resistant nail varnish on the enamel samples was carefully removed using acetone in both lingual and buccal surfaces, and SEM-EDX analyses were performed.

Samples were air-dried, mounted on aluminum stubs, coated with a 10 nm gold layer to ensure conductivity, and then observed using TESCAN VEGA 3 LMU SEM (Center for Electron Microscopy-CISMIN Department of SIMAU, Università Politecnica delle Marche, Ancona, Italy). SEM images were acquired to investigate the morphology of enamel at different magnifications: 500× and 1000×. The operating parameters were operating at an accelerating voltage of 15 kV, working distance of 10 mm, and secondary electron (SE) detector.

The chemical surface characterization was performed by means of EDX using EDAX Element Microanalysis (AMETEK Gmbh, EDAX Business Unit, Weiterstadt, Germany).

Each sample was divided into distinct regions (sound, demineralized, and treated). For each region, three EDX spectra were acquired from randomly selected fields. The three readings were averaged to obtain a single mean value per tooth per condition, which represented the experimental unit for statistical analysis. Field-level data were not treated as independent replicates to avoid pseudoreplication. The following operating parameters were considered: working distance of 15 mm, acceleration voltage of 25 kV, and 500× magnification. EDX spectra were acquired with a dwell time of 60 s. Quantification was performed using ZAF correction to account for atomic number, absorption, and fluorescence effects.

The degree of remineralization was assessed by measuring the amount (in atomic %) of phosphorus (P) and calcium (Ca) and calculating their ratio (Ca/P) in the treated samples. Results were reported as mean value and standard deviation.

The operator performing SEM-EDX analyses was blinded to group and time allocation to minimize measurement bias. Data labeling and file codes were anonymized before image capture and EDX quantification.

### 2.6. Statistical Analysis

All data were analyzed using statistical software Prism8 (GraphPad Software, Version 10.4.1, CA, USA). Descriptive statistics were calculated and reported as mean ± standard deviation. A two-way independent measures ANOVA was performed to evaluate the effects on enamel Ca/P ratio of the type of remineralizing agent and the time of application. When significant differences were found, post hoc multiple comparisons were performed using Bonferroni test to adjust for multiple comparisons. The significance level was set at α = 0.05. Differences between groups were considered statistically significant when the 95% confidence interval (CI) of the mean difference did not include zero and the adjusted *p*-value was below 0.05. Bonferroni correction was applied to adjust *p*-values for all post hoc multiple comparisons.

Sample size calculation was conducted using G*Power 3.1 (Franz Faul, Kiel University, Kiel, Germany) to determine the minimum sample size for a two-way independent-measures ANOVA (factors: remineralizing agent × time (7, 14, 21, and 28 days)), assuming a large effect size for the interaction (Cohen’s f = 1.32), α = 0.05, and power (1 − β) = 0.80, based on preliminary data and previous studies [20,21,26]. The calculation targeted the interaction effect (agent × time), which represents the primary endpoint of the study. Observed effect sizes from the final ANOVA confirmed this assumption (partial η^2^ = 0.635 for the agent × time interaction, corresponding to f = 1.32). With three treatment groups and four time points, the required total sample size was 20 teeth for each group (n = 5 per time point).

## 3. Results

**Figure 2:**
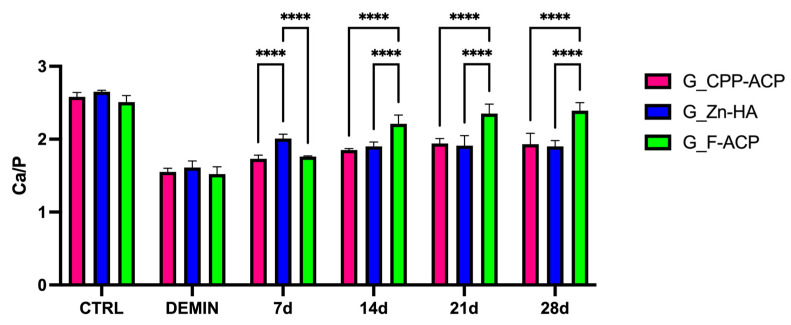
Bar graph showing the mean Ca/P ratio (±standard deviation) of enamel samples from the three experimental groups at different time points (7, 14, 21, and 28 days): G_CPP-ACP (casein phosphopeptide–amorphous calcium phosphate), G_Zn-HA (zinc-hydroxyapatite), and G_F-ACP (fluoridated amorphous calcium phosphate). The CTRL group represents sound enamel (untreated), while DEMIN indicates demineralized enamel prior to remineralization treatment. Asterisks denote statistically significant differences between treatment groups at each time point. Post hoc Bonferroni-adjusted comparisons at each time point: **** *p* < 0.0001, number = 5 per time point per agent.

**Figure 3:**
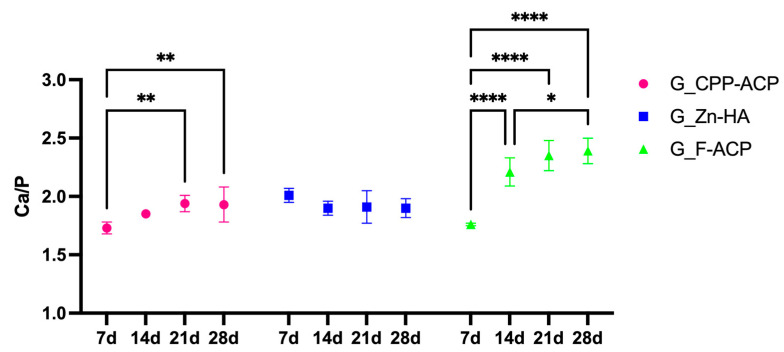
Time-dependent variation of the Ca/P ratio (mean values) of demineralized enamel surfaces treated with three different remineralizing agents: G_CPP-ACP (casein phosphopeptide–amorphous calcium phosphate), G_Zn-HA (zinc-hydroxyapatite), and G_F-ACP (fluoridated amorphous calcium phosphate), over a 28-day period. Measurements were performed by SEM-EDX at 7, 14, 21 and 28 days post-treatment. Asterisks denote statistically significant differences intra group between each time point. Post hoc Bonferroni-adjusted comparisons at each time point: * *p* < 0.05; ** *p* < 0.001; **** *p* < 0.0001, n = 5 per time point per agent.

The Ca/P ratio values, obtained from SEM-EDX analysis, are presented in Figure 2 and Figure 3. No statistically significant difference was identified in terms of CTRL and DEMIN between the different time points within each group (*p* > 0.05). Therefore, Figure 2 shows the average Ca/P value (calculated on 20 samples) for each group.

A significant reduction in the Ca/P ratio was observed among all groups following the demineralization protocol (DEMIN), in comparison to sound enamel (CTRL). This outcome serves as a confirmation of the effective induction of caries-like lesions.

Following the application of remineralizing agents, progressive increases in the Ca/P were observed over time in all treatment groups.

At 7 days, G_Zn-HA exhibited significantly higher Ca/P values in comparison to both G_CPP-ACP (*p* < 0.05) and G_F-ACP (*p* < 0.05). However, no significant difference was observed between G_CPP-ACP and G_F-ACP. Furthermore, at day 14, G_F-ACP exhibited a significantly greater remineralization effect in comparison to both G_CPP-ACP and G_Zn-HA (*p* < 0.05). This trend persisted through days 21 and 28, suggesting a superior remineralization potential of the F-ACP-based agent, compared with the other groups.

The comparison with CTRL and DEMIN values highlights that G_F-ACP progressively approached the Ca/P ratio of healthy enamel (CTRL) by day 21, while G_CPP-ACP and G_Zn-HA remained at lower levels (Figure 1).

The time-dependent remineralization response is also illustrated in Figure 3, where G_F-ACP demonstrated a good remineralization increase between 7 and 14 days (*p* < 0.05). This was followed by a plateau phase, during which no statistically significant difference was evident between 14 and 21 days (*p* > 0.05). Then, a further phase of remineralization occurred between 21 and 28 days (*p* < 0.05). G_CPP-ACP reached a plateau after 21 days of treatment, in which Ca/P value was statistically different between the one at 7 days (*p* < 0.05). On the contrary, in G_Zn-HA, Ca/P values highlighted no statistically significant differences between the different timing points (*p* > 0.05).

A summary of the mean ± SD Ca/P ratios for each treatment group at the different observation periods is reported in Table 2. This table facilitates comparison of time-dependent mineral gain and complements the graphical representation in Figure 2 and Figure 3.

**Figure A1:**
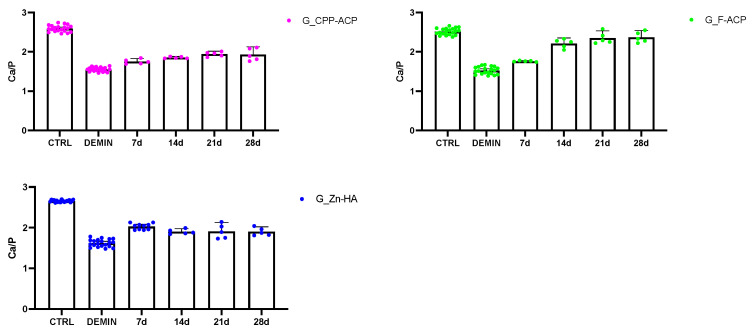
Distribution of Ca/P ratio values for each remineralizing agent (CPP-ACP, Zn-HA, and F-ACP) across all observation times (7, 14, 21, and 28 days). Each dot represents the mean Ca/P ratio per tooth, calculated as the average of three EDX spectra acquired from randomly selected fields within each region (sound, demineralized, and treated). Bars indicate mean ± standard deviation (SD), and error bars represent the 95% confidence intervals of the mean. Control (CTRL) and demineralized (DEMIN) enamel regions are shown as intra-sample references. This figure illustrates within-group variability and complements Figure 2 and Figure 3 in the main text, providing transparency on data distribution and confidence intervals.

Individual Ca/P ratio distributions and 95% CI for all treatment groups and time points are provided in Figure A1, which illustrates the variability among samples and supports the statistical findings reported in the main text.

Bonferroni multiple comparisons for each time point are reported in Table A1 (Appendix A), confirming that Zn-HA showed higher Ca/P ratios than the other agents at 7 days, whereas F-ACP exhibited significantly higher Ca/P than both CPP-ACP and Zn-HA from 14 days onward.

**Figure 4:**
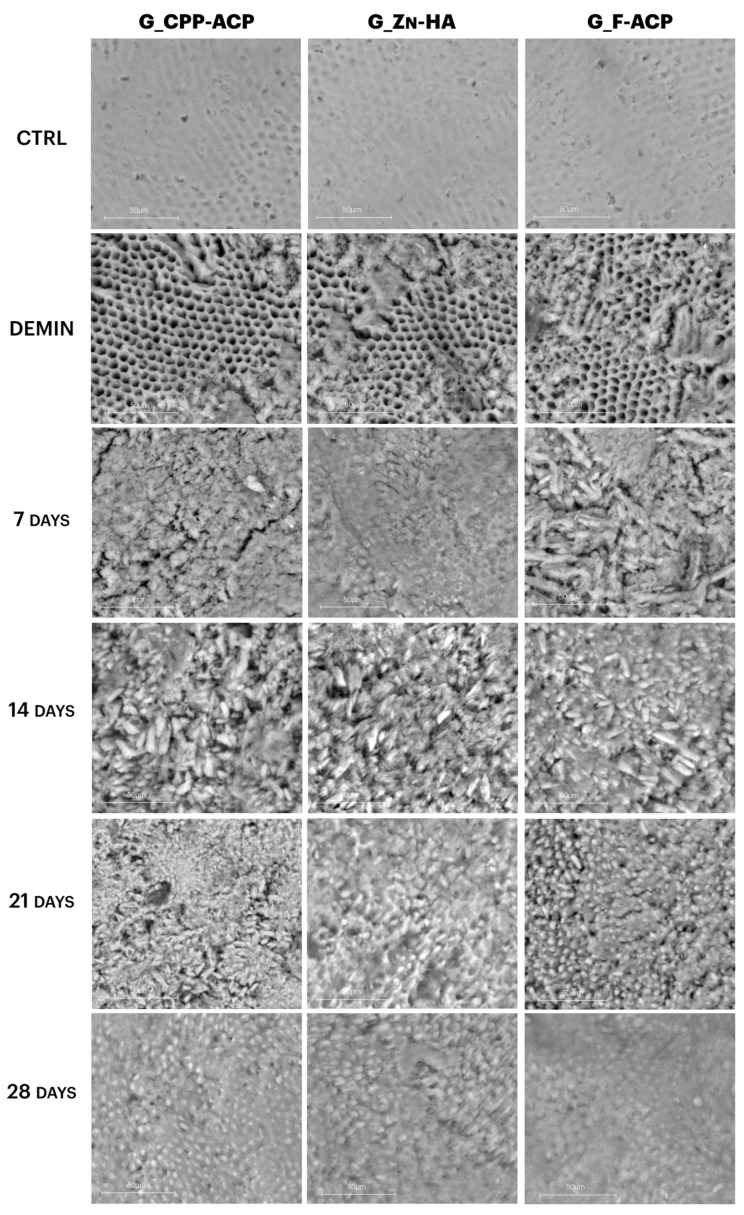
Representative scanning electron micrographs of enamel surfaces (sound, demineralized and post-treatment with CPP-ACP, Zn-HA, F-ACP), at different time points, 7, 14, 21, and 28 days, (number = 5 per time). All images acquired at 50 µm magnification. Demineralized surfaces (DEMIN) exhibit increased porosity and loss of enamel structure, while progressive surface recovery is evident over time. G_F-ACP shows the most homogeneous and organized surface at 28 days, resembling the microstructure of sound enamel.

Representative scanning electron micrographs of enamel surfaces at 50 µm magnification are shown in Figure 4. In the CTRL (sound enamel) samples, the enamel prisms appear smooth, compact, and well-organized, with a typical rod-like structure and no surface discontinuities.

After demineralization (DEMIN), all groups displayed clear surface damage characterized by porosity, irregular texture, and loss of prism architecture, consistent with subsurface lesion formation.

In G_CPP-ACP, progressive surface recovery was observed over time. At 7 and 14 days, the enamel surface remained irregular, though some granular deposits were visible. By 21 and 28 days, a partial reorganization of surface texture was observed, but the enamel remained less compact than in CTRL.

G_Zn-HA showed earlier signs of surface coverage, with amorphous deposits appearing as early as 7 days. However, the surface remained heterogeneous, and full prism reorganization was not evident even at 28 days.

In contrast, G_F-ACP demonstrated a more uniform and denser mineral layer beginning at 14 days, which progressively thickened and smoothed by day 28. At the final time point, G_F-ACP showed the most homogeneous surface, closely resembling the original CTRL enamel microstructure.

## 4. Discussion

The present study evaluated the remineralization efficacy of three professional agents (CPP-ACP, Zn-HA, and F-ACP) on artificially induced enamel lesions, using SEM and EDX analysis across four time-points (7, 14, 21 and 28 days).

A key finding was the time-dependent nature of enamel remineralization. All three agents demonstrated a gradual increase in remineralization over time, as reflected by both SEM and EDX results. Pronounced improvements were consistently observed at 7 and 14 days, whereas the 21 and 28 days led to moderate changes in Ca/P ratio. This highlighted the importance of sustained application in maximizing remineralizing effects, particularly for biomimetic agents like F-ACP. These findings were consistent with the scientific literature supporting prolonged or repeated topical applications to enhance clinical efficacy [21,27].

The extent and pattern of remineralization differed substantially among the three formulations, probably due to the structural and chemical characteristics of the materials. In particular, F-ACP based agent emerged as the most effective agent in this study, exhibiting the highest Ca/P values from day 14 onwards, eventually approaching those of sound enamel (CTRL) by day 21. Its dual mechanism, fluoride-enhanced acid resistance and amorphous calcium phosphate reservoir, enables deeper penetration and robust mineral recovery, consistent with findings by previous authors [28,29]. Studies have reported that F-ACP outperforms both CPP-ACP and Zn-HA in restoring optical properties and reducing lesion porosity [29,30]. Moreover, the incorporation of fluoride into ACP enhances crystal maturation and stability, offering a long-lasting protective effect [28].

G_CPP-ACP acts as a reservoir of calcium and phosphate ions, stabilizing them in amorphous form and releasing them at the tooth surface, facilitating the nucleation of hydroxyapatite [14,31]. It demonstrated a moderate but consistent remineralizing potential over time. This aligned with previous studies, highlighting its capacity to deposit calcium and phosphate in early carious lesions and enhance enamel surface reconstitution [31,32]. However, its effect tends to plateau between 14 and 21 days and a recent systemic review suggests that CPP-ACP treatment is only effective in the early stages of enamel lesion due to its limited ability to penetrate [33,34].

G_Zn-HA mimics enamel structure and enables ion exchange and new formation of HA crystals [35] and displayed the lowest trend of remineralization respect to the other groups at each time points, reaching a plateau after 7 seven days of treatment. The surface morphology recovery of this agent presented a peak at 7 days probably due to its biomimetic crystalline structure, with the deposition on synthetic HA on demineralized enamel micropores, reducing lesion depth and enhancing mineral gain [20]. The plateau effect seen in the groups suggests that remineralization efficacy may stabilize with time, depending on agent properties and delivery mechanisms.

SEM observations supported EDX quantitative findings: while all treatments induced partial surface recovery, G_F-ACP produced the most homogeneous and compact mineral layer with well-defined prism structures by day 21. G_CPP-ACP showed granular deposits and gradual surface reorganization, whereas G_Zn-HA exhibited heterogeneous mineral coverage without full structural restoration. These morphological differences aligned with the distinct mechanisms of action: F-ACP facilitates rapid fluorapatite formation due to its combined fluoride and calcium-phosphate content [15], while CPP-ACP relies on sustained ion release mediated by casein phosphopeptides [13], and Zn-HA promotes enamel repair primarily via biomimetic deposition [10].

From a clinical perspective, CPP-ACP, Zn-HA, and F-ACP are all indicated for the non-invasive management of early enamel lesions, hypersensitivity, and post-orthodontic white spot lesions. While CPP-ACP and Zn-HA act primarily through calcium–phosphate ion release and surface adsorption, F-ACP additionally provides fluoride-mediated nucleation and stabilization of apatite phases.

The present findings suggest that, under controlled in vitro conditions, F-ACP may achieve faster and more complete surface remineralization—approaching the Ca/P ratio of sound enamel within 21 days, compared with CPP-ACP and Zn-HA, which reached partial recovery earlier and plateaued. However, this behavior reflects only surface-level compositional recovery, neither full subsurface remineralization nor mechanical restoration.

CPP-ACP and Zn-HA, while less effective in this study, still demonstrated notable remineralization capacity and may serve as valuable adjuncts in preventive dentistry, particularly for patients contraindicated for fluoride or seeking biomimetic, non-fluoride alternatives.

The obtained results indicated a strong interaction between treatment type and time. From a clinical perspective, these findings suggest that a minimum of approximately 21 consecutive days of daily application may be required to achieve optimal remineralization effects, as observed for F-ACP. However, patient compliance and individual salivary conditions are critical determinants of clinical efficacy and should be considered in practical protocols.

Despite promising results, the present study has limitations: it is an in vitro model and does not simulate factors such as salivary flow, biofilm activity, and patient compliance, as well as the overall biological complexity of the oral environment, which influence remineralization dynamics in vivo. The absence of saliva, pellicle formation, biofilm activity, and masticatory forces may alter ion diffusion and mineral deposition, influencing remineralization kinetics. Another limitation of the present investigation is the exclusive use of SEM-EDX for surface and compositional evaluation. Complementary analyses such as microhardness testing, nanoindentation, and micro-CT could provide insights into the mechanical reinforcement and depth of remineralization. Therefore, future clinical trials are necessary to confirm the present findings and to assess the long-term performance of these agents under dynamic oral conditions.

The present results, derived from surface Ca/P ratios and SEM morphology, indicate a clear compositional recovery at the enamel surface, although EDX analysis is limited to surface elemental quantification and does not provide information on subsurface mineralization depth or mechanical properties. Complementary analyses such as microhardness test and micro-CT could provide insights into the mechanical reinforcement and depth of remineralization. This combined analytical approach would allow a more complete characterization of enamel recovery at both microstructural and functional levels.

Nonetheless, this study provides a strong foundation for the selection and application of remineralizing agents in preventive and restorative dentistry, suggesting that the time of application represents a key factor for clinical success.

## 5. Conclusions

The present in vitro study demonstrated that all three professional agents (CPP-ACP, Zn-HA, and F-ACP) exhibited a time-dependent remineralization effect on artificially demineralized enamel. Among the examined products, F-ACP demonstrated the most significant remineralizing efficacy after 21 days, as indicated by the Ca/P ratio and the observation of enhanced surface morphological integrity via SEM-EDX analysis. Zn-HA and CPP-ACP were highly effective agents, displaying promising remineralization behavior, with a moderate but consistent activity over time. However, these findings reflect surface compositional changes and should be interpreted with caution until validated in clinical settings. The data support the potential of biomimetic remineralizing agents as adjuncts to daily preventive care, encouraging future translational research. Thus, further studies employing mechanical and cross-sectional assessments are required to confirm the full extent of remineralization.

The findings emphasize the central function of the time in achieving effective remineralization effects. Prolonged and continuous exposure to these agents has been demonstrated to result in a more substantial mineral gain, thereby supporting long-term treatment strategies for non-cavitated carious lesions. These outcomes are clinically relevant in the fields of preventive and minimally invasive dentistry, especially for patients with early enamel lesions or those requiring alternatives to fluoride-based therapies.

Beyond the specific efficacy of the tested agents, this study makes a significant contribution to the broader field of dental biomaterials research. The quest for novel remineralizing agents that combine bioactivity, biocompatibility, simplicity of clinical application and specific clinical protocols remains nowadays a key priority.

While the present findings highlight the superior remineralization potential of F-ACP, they should be interpreted within the limits of the in vitro model. Further in vivo and clinical studies are necessary to validate these outcomes under real oral conditions.

## Figures and Tables

**Table 1:** Chemical composition of the tested materials.

Material	Manufacturer	Active Ingredient	Other Ingredients
GC Tooth Mousse (G_CPP-ACP)	Recaldent Europe	casein phosphopeptide–amorphous calcium phosphate (CPP-ACP)	Pure water, glycerol, casein phosphopeptide–amorphous calcium–phosphate, D-sorbitol, sodium carboxymethyl cellulose, propylene glycol, silicon dioxide, titanium dioxide, xylitol, phosphoric acid, flavoring, zinc oxide, sodium saccharin, ethyl p-hydroxybenzoate, magnesium oxide, guar agam, propyl p-hydroxybenzoate, butyl p-hydroxybenzoate.
Biorepair Desensitizing Enamel Repairer (G_Zn-HA)	Coswell Spa, Italy	Zinc-hydroxyapatite (Zn-HA)	Water, zinc hydroxyapatite, hydrated silica, silica, sodium myristoyl sarcosinate, sodium methyl cocoyl taurate, sodium bicarbonate, aroma, sodium saccharin, phenoxyethanol, benzyl alcohol, sodium benzoate, citric acid, menthol.
Biosmalto (G_F-ACP)	Curasept Spa, Italy	Fluoridated amorphous calcium phosphate (F-ACP), 1450 ppm F^-^	Glycerin, PEG-8, silica, strontium acetate, calcium phosphate carbonate citrate fluoride, hydroxypropylcellulose, xylitol, acrylates/C10-30 alkyl acrylate crosspolymer, PEG-40 hydrogenated castor oil, sodium hyaluronate, potassium acesulfame, p-anisic acid, aroma, sodium hydroxide.

**Table 2:** Ca/P ratio (atomic%) for each remineralizing agent (CPP-ACP, Zn-HA, F-ACP) across time (7, 14, 21, and 28 days). Data are presented as mean and standard deviation (SD), calculated from five teeth per group and time point, with each value representing the average of three EDX spectra acquired from randomly selected fields within each region.

	G_CPP-ACP		G_Zn-HA		G_FACP	
	*Mean*	*SD*	*Mean*	*SD*	*Mean*	*SD*
**CTRL**	2.59	0.08	2.65	0.02	2.51	0.08
**DEMIN**	1.55	0.05	1.62	0.09	1.52	0.1
**7d**	1.75	0.06	2.03	0.07	1.76	0.01
**14d**	1.85	0.02	1.9	0.06	2.21	0.11
**21d**	1.94	0.06	1.91	0.17	2.35	0.14
**28d**	1.93	0.15	1.9	0.1	2.37	0.13

## Data Availability

The data presented in this study are available on request from the corresponding author.

## Appendix A

**Table A1:** Bonferroni-adjusted post hoc comparisons of Ca/P ratios among remineralizing agents (CPP-ACP, Zn-HA, and F-ACP) at each observation time point. Asterisks denote statistically significant differences: **** = *p* < 0.0001; ns = not statistically significant.

Bonferroni’s Multiple Comparisons Test	Predicted (LS) Mean Diff	95.00% CI of Diff	Summary	Adjusted *p* Value
** 7d**				
G_CPP-ACP vs. G_Zn-HA	−0.2800	−0.3935 to −0.1665	****	<0.0001
G_CPP-ACP vs. G_F-ACP	−0.01000	−0.1410 to 0.1210	ns	>0.9999
G_Zn-HA vs. G_F-ACP	0.2700	0.1565 to 0.3835	****	<0.0001
** 14d**				
G_CPP-ACP vs. G_Zn-HA	−0.05000	−0.1810 to 0.08104	ns	>0.9999
G_CPP-ACP vs. G_F-ACP	−0.3600	−0.4910 to −0.2290	****	<0.0001
G_Zn-HA vs. G_F-ACP	−0.3100	−0.4410 to −0.1790	****	<0.0001
** 21d**				
G_CPP-ACP vs. G_Zn-HA	0.03200	−0.09904 to 0.1630	ns	>0.9999
G_CPP-ACP vs. G_F-ACP	−0.4100	−0.5410 to −0.2790	****	<0.0001
G_Zn-HA vs. G_F-ACP	−0.4420	−0.5730 to −0.3110	****	<0.0001
** 28d**				
G_CPP-ACP vs. G_Zn-HA	0.03000	−0.1010 to 0.1610	ns	>0.9999
G_CPP-ACP vs. G_F-ACP	−0.4400	−0.5710 to −0.3090	****	<0.0001
G_Zn-HA vs. G_F-ACP	−0.4700	−0.6010 to −0.3390	****	<0.0001
